# Bioactivity Assessment of Functionalized TiO_2_ Powder with Dihydroquercetin

**DOI:** 10.3390/ijms26041475

**Published:** 2025-02-10

**Authors:** Valentina Nikšić, Andrea Pirković, Biljana Spremo-Potparević, Lada Živković, Dijana Topalović, Jovan M. Nedeljković, Vesna Lazić

**Affiliations:** 1 Vinča Institute of Nuclear Sciences—National Institute of the Republic of Serbia, Centre of Excellence for Photoconversion, University of Belgrade, 11351 Belgrade, Serbia; valentina.niksic@vin.bg.ac.rs (V.N.); jovned@vin.bg.ac.rs (J.M.N.); 2 Department for Biology of Reproduction, INEP Institute for Application of Nuclear Energy, University of Belgrade, 11080 Belgrade, Serbia; andrea.pirkovic@inep.co.rs; 3 Department of Pathobiology, Faculty of Pharmacy, University of Belgrade, 11221 Belgrade, Serbia; bilja22@pharmacy.bg.ac.rs (B.S.-P.); lada@pharmacy.bg.ac.rs (L.Ž.);

**Keywords:** TiO_2_, dihydroquercetin, interfacial charge transfer complex, cytotoxicity, antigenotoxicity, antimicrobial activity

## Abstract

Biological activities, including cell viability, oxidative stress, genotoxicity/antigenotoxicity, and antimicrobial activity, were evaluated for a visible-light-responsive TiO_2_-based ICT complex with dihydroquercetin (DHQ) and compared with pristine TiO_2_, its inorganic component. Pristine TiO_2_ did not induce cytotoxicity in MRC-5 or HeLa cells within the tested concentration range (1–20 mg/mL), while TiO_2_/DHQ displayed a significant reduction in cell viability in both cell lines at higher concentrations (≥10 mg/mL). The analysis of reactive oxygen species (ROS) production revealed that TiO_2_/DHQ significantly reduced ROS levels in both cell types (MRC-5 and HeLa), with HeLa cells showing a more substantial reduction at lower concentrations. Genotoxicity assessment using the comet assay demonstrated that TiO_2_ induced DNA damage in MRC-5 cells, while TiO_2_/DHQ did not, indicating that DHQ mitigates the genotoxic potential of TiO_2_. Furthermore, TiO_2_/DHQ exhibited antigenotoxic effects by reducing H_2_O_2_-induced DNA damage in MRC-5 cells, supporting its protective role against oxidative stress. Preliminary antimicrobial tests revealed that TiO_2_/DHQ exhibits antimicrobial activity against *E. coli* under visible-light excitation, while TiO_2_ does not. These findings suggest that the TiO_2_-based ICT complex with DHQ with enhanced antioxidant properties can potentially serve as a safe, non-toxic biocide agent.

## 1. Introduction

Titanium dioxide (TiO_2_) is a widely studied, wide-bandgap (3.2 eV) photocatalyst [1], displaying antimicrobial activity when exposed to ultraviolet (UV) light. Photogenerated charge carriers (electrons and holes) lead to the formation of reactive oxygen species (superoxide anions and hydroxyl radicals, respectively) capable of effectively inactivating a broad range of microorganisms, including bacteria, viruses, and fungi [2]. Because of that, TiO_2_ is a promising candidate for a variety of eco- and bio-related applications, such as wastewater and air purification [1], self-cleaning surfaces [3], medical devices [4,5], and food packaging [6]. For example, TiO_2_ coatings can reduce the risk of healthcare-associated infections in hospitals [7], while wastewater treatment with TiO_2_-based materials leads to the photocatalytic degradation of organic pollutants and the inactivation of pathogens [8]. However, excitation by high-energy photons (UV light) limits any photo-driven application of TiO_2_, including antimicrobial. So, an extension of TiO_2_’s response in the visible spectral range (Vis) is crucial from a technological point of view, particularly in indoor and low-light-intensity environments. The most recent promising strategy to obtain visible-light-responsive TiO_2_-based composites is the formation of interfacial charge transfer (ICT) complexes upon coordination of organic molecules [9], preferably benzene derivatives, to the TiO_2_ surface [10]. The functionalization of the TiO_2_ surface with properly chosen non-absorbing in Vis organic molecules leads to red-shifted TiO_2_ absorption, enabling its photoactivity under visible-light excitation [11]. Recent studies have demonstrated the successful formation of TiO_2_-based ICT complexes with various ligand molecules (catechol [10,12,13,14], salicylic acid [10,14,15], and phenol [16,17,18,19] derivatives). ICT complexes with catecholate- and salicylate-type ligands are stable when exposed to light and display enhanced photocatalytic abilities [13,14,20,21,22,23]. On the other hand, when phenol and its derivatives are organic components of the ICT complex, their degradation [16,17,18,19] occurs under Vis light [24,25].

However, TiO_2_ particulates can pass into an organism via oral, inhalation, and dermal exposure routes and accumulate in various tissues. Previous research on human lung cell systems (A549 epithelial cells, macrophages differentiated from THP-1, and A549/differentiated THP-1 cocultures) indicated that the morphological and interface properties of TiO_2_ crucially affect its toxicity [26]. Also, exposure to TiO_2_ NPs in human embryonic lung cells resulted in cytotoxicity and genotoxicity after 24 h of exposure, suggesting that exposure to TiO_2_ NPs may lead to undesired health effects and bioaccumulation [27].

In the most recent studies, high-biological-value compounds originating from plants, such as flavonoids, have gained attention since, on the one hand, they can coordinate on metal-oxide surfaces and modulate their optical properties and, on the other hand, they can diminish the undesired toxic effect of inorganic particulates [28,29], which increases with decreasing particle size. However, studies concerning the use of bioactive compounds to facilitate the formation of ICT complexes and take advantage of their enhanced optical properties in photocatalytic bactericidal activity [30], as well as to evaluate toxicity using either in vitro [31,32] or in vivo [33,34] experiments upon particle functionalization, are still scarce.

Dihydroquercetin (DHQ), a naturally occurring flavonoid, is an efficient scavenger of reactive oxygen species (ROS) capable of protecting cells from oxidative stress, a crucial factor in various diseases and aging processes [35]. Because of this, DHQ has a wide range of therapeutic effects, including anticancer, anti-inflammatory, antigenotoxic, anti-Alzheimer, antiangiogenic, and diuretic properties, as well as being effective in the prevention of diabetes and some cardiovascular diseases [36,37].

Very recently, we prepared a TiO_2_-based ICT complex with DHQ (TiO_2_/DHQ), performing thorough optical characterization supported with density functional theory (DFT) calculations and indirect electron paramagnetic resonance (EPR) spectroscopy techniques to detect and identify transient species upon photoexcitation [29]. As an extension, to follow the safer-by-design approach, we evaluated the cytotoxic and genotoxic potential of TiO_2_/DHQ in human fetal lung fibroblasts MRC-*5* in vitro, a cell type often used in cytocompatibility studies [38]. Also, knowing that TiO_2_/DHQ displays extended absorption in the Vis spectral range, the antimicrobial efficiency of TiO_2_/DHQ against *E. coli* was tested using solely Vis light to estimate the possibility of its safe application in antimicrobial treatments.

## 2. Results

### 2.1. Basic Properties of ICT Complex Between TiO_2_ and DHQ

The composition of the TiO_2_/DHQ sample was determined by analyzing the TG curve (Figure S1 in Supplementary Materials). The gradual decrease in weight of ~1% is due to the removal of adsorbed water. Then, melting, followed by the decomposition of DHQ, takes place in the temperature range of 230–400 °C, and finally, at the high-temperature end of the TG curve, the residual mass of ~95% corresponds to the remaining TiO_2_, indicating that the DHQ content in TiO_2_/DHQ is ~4%.

**Figure 1:**
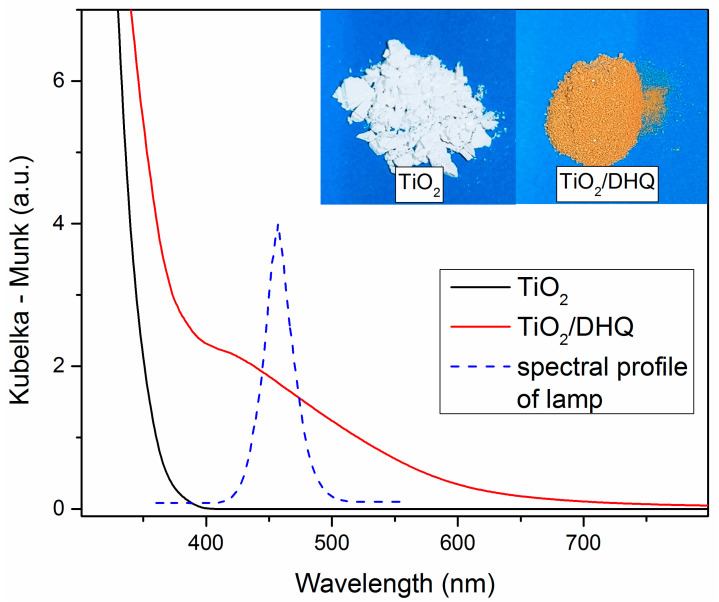
Kubelka–Munk transformations of DRS data for the pristine TiO_2_ (Aeroxide^®^ P25) powder and TiO_2_-based ICT complex with DHQ. Also, images of TiO_2_ and TiO_2_/DHQ and the spectral profile of emitted light used in antimicrobial tests are included.

A prerequisite for understanding the toxicological and antimicrobial behavior of functionalized TiO_2_ powder with DHQ lies in its optical properties and surface structure, i.e., the coordination of DHQ to the TiO_2_ surface. Figure 1 shows the optical absorption of pristine TiO_2_ powder (Degussa P25) and the corresponding ICT complex with DHQ, obtained by Kubelka–Munk transformations of diffuse reflection spectroscopy (DRS) data. Also, images of pristine TiO_2_ and TiO_2_/DHQ powders and the spectral profile of excitation light from the light source used in antimicrobial testing are included in Figure 1. The significant red shift in the absorption onset in TiO_2_/DHQ (~600 nm) is a consequence of the formation of the ICT complex and is consistent with the reported literature data for TiO_2_-based ICT complexes with catecholate-type ligands [10,11,12,13,14].

### 2.2. Cytotoxicity

**Figure 2:**
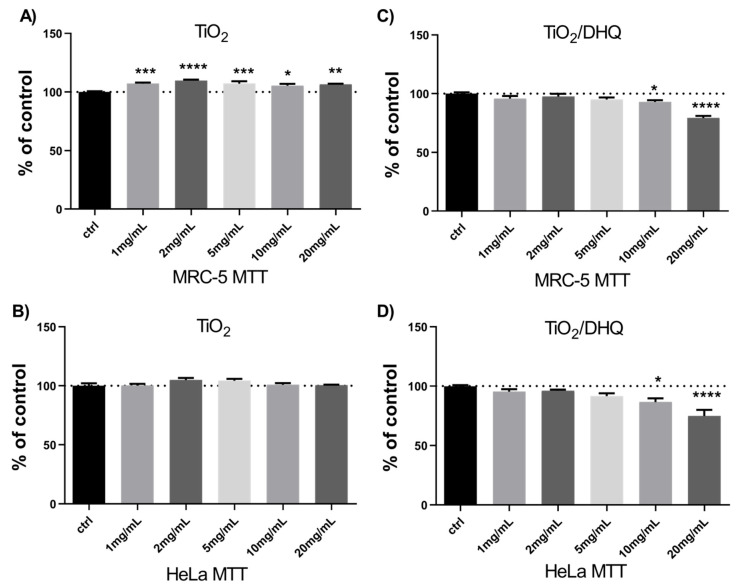
(**A**,**B**): Cytotoxicity of TiO_2_ at a range of concentrations (1, 2, 5, 10, and 20 mg/mL) determined by MTT assay in MRC-5 cells (**A**) and HeLa cells (**B**), expressed as a percentage out of the control. (**C**,**D**): Cytotoxic effect of TiO_2_/DHQ at a range of concentrations (1, 2, 5, 10, and 20 mg/mL) in MRC-5 cells (**C**) and HeLa cells (**D**), determined by MTT assay. The data are expressed as mean + SEM. * *p* < 0.05, ** *p* < 0.01, *** *p* < 0.001, **** *p* < 0.0001 versus control (by one-way Analysis of Variance (ANOVA) with Tukey’s multiple comparison post hoc test).

The evaluation of the effects of 24 h incubation with pristine TiO_2_ on cell viability in MRC-5 cells (Figure 2A) and HeLa cells (Figure 2B) showed that TiO_2_, in the investigated concentration range (1–20 mg/mL), did not induce cytotoxic effects. Moreover, a slight increase in the percentage of viable non-malignant MRC-5 cells was observed compared to the control sample, while in the case of HeLa cancer cells, there was no significant difference versus the non-treated control. The concentration-dependent cell viability in MRC-5 and HeLa cells after 24 h of incubation with TiO_2_/DHQ is presented in Figure 2C and Figure 2D, respectively. The results indicate that the exposure of MRC-5 cells to the released material from TiO_2_/DHQ leads to significant cytotoxic effects at concentrations above 10 mg/mL compared to the control (sample treated only with medium). However, the observed percentage reduction in viable cells after the treatment with 10 and 20 mg/mL of TiO_2_/DHQ was quite small (7.0% and 21.6% viability reduction, respectively). Similarly, the exposure of HeLa cells to the released material from TiO_2_/DHQ over 24 h displayed concentration-dependent cytotoxicity (Figure 2D) and a significant reduction in viability at a 10 mg/mL concentration or higher (compared to the control). The most pronounced decrease in viability (25%) was observed for the highest concentration of TiO_2_/DHQ (20 mg/mL).

**Figure 3:**
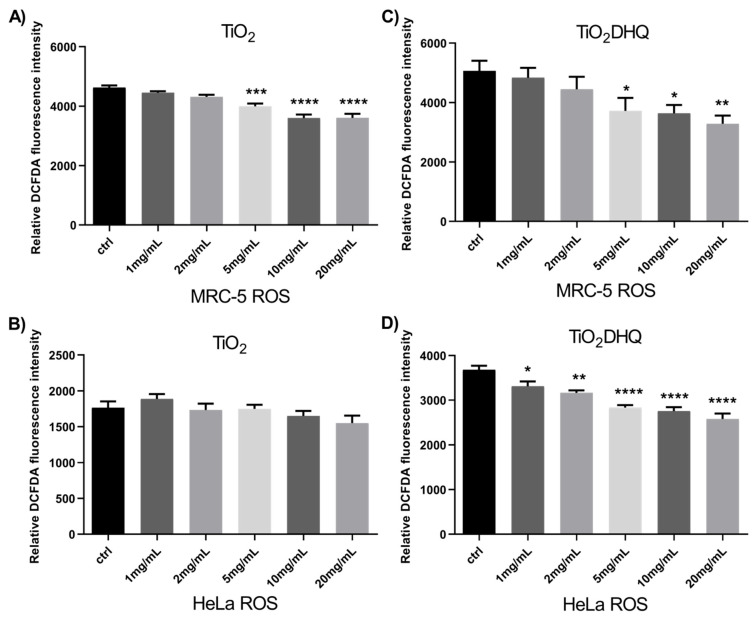
(**A**,**B**): The effect of TiO_2_ at a range of concentrations (1, 2, 5, 10, and 20 mg/mL) on the production of reactive oxygen species (ROS) in MRC-5 cells (**A**) and HeLa cells (**B**), determined by the H2DCFDA assay, expressed as relative fluorescence intensity. (**C**,**D**): The effect of TiO_2_DHQ on the production of reactive oxygen species (ROS) at a range of concentrations (1, 2, 5, 10, and 20 mg/mL) in MRC-5 cells (**C**) and HeLa cells (**D**), determined by the H2DCFDA assay. The data are expressed as mean + SEM. * *p* < 0.05, ** *p* < 0.01, *** *p* < 0.001, **** *p* < 0.0001 versus control (by one-way Analysis of Variance (ANOVA) with Tukey’s multiple comparison post hoc test).

Figure 3 shows the production of ROS in MRC-5 and HeLa cells as a function of the concentration of pristine TiO_2_ and the TiO_2_-based ICT complex with DHQ after the 24 h incubation period. The analysis of the obtained results indicated a decrease in ROS production in non-malignant MRC-5 cells with the increase in the pristine TiO_2_ concentration compared to the control (Figure 3A), with a pronounced effect for the highest TiO_2_ concentration (≥5 mg/mL). In the case of HeLa cancer cells, none of the applied TiO_2_ concentrations influenced ROS production compared to the control (Figure 3B). On the other hand, in both cell lines, MRC-5 and HeLa, the impact of TiO_2_/DHQ treatment on ROS levels was significant (Figure 3C and Figure 3D, respectively). The incubation of MRC-5 cells with TiO_2_/DHQ induced a significant reduction in the ROS level when cells were exposed to a TiO_2_/DHQ concentration equal to or higher than 5 mg/mL, while in the case of HeLa cells, a concentration-dependent reduction in the ROS level started from the lowest TiO_2_/DHQ concentration (1 mg/mL).

### 2.3. Genotoxicity and Antigenotoxicity

**Figure 4:**
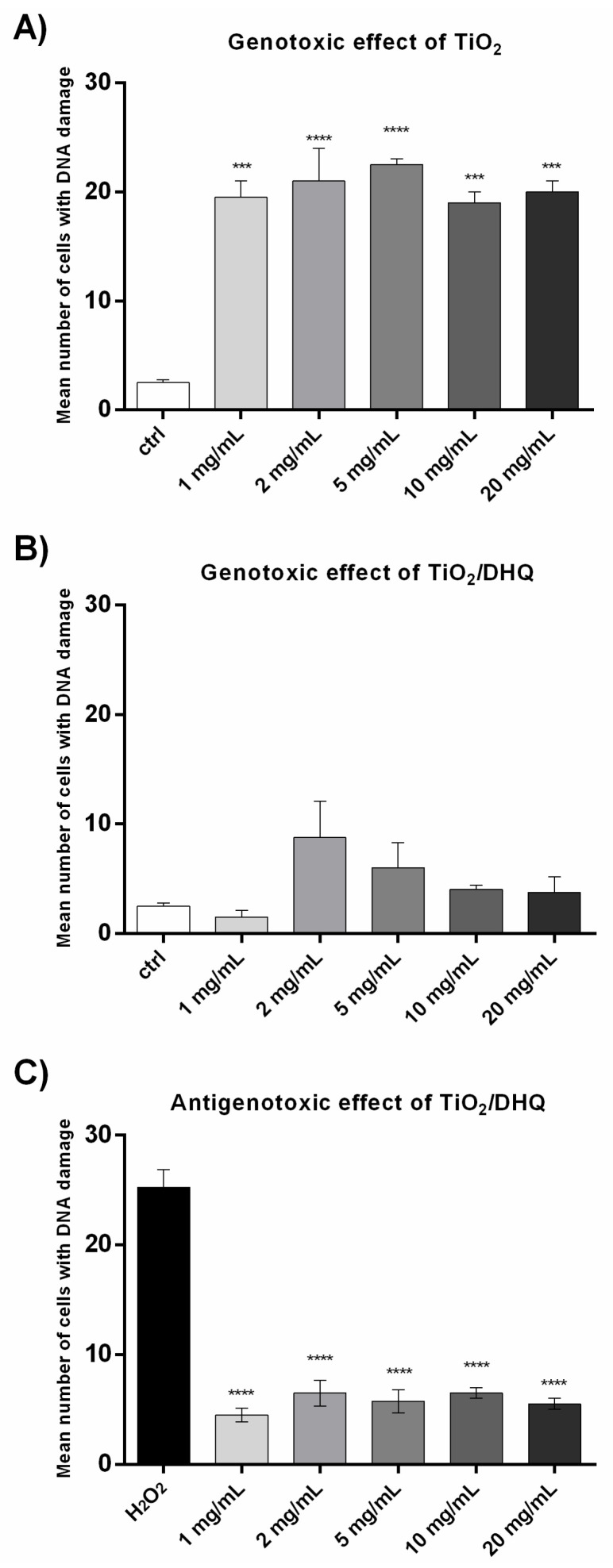
Genotoxic effect of (**A**) TiO_2_ and (**B**) TiO_2_/DHQ in MRC-5 cells and (**C**) antigenotoxic effect of TiO_2_/DHQ against DNA damage induced by H_2_O_2_ in MRC-5 cells at a range of concentrations (1, 2, 5, 10, and 20 mg/mL), determined by the comet assay. The data are expressed as the mean number of cells with DNA damage + SEM. *** *p* < 0.001, **** *p* < 0.0001 versus control (by one-way Analysis of Variance (ANOVA) with Tukey’s multiple comparison post hoc test).

Besides their cytotoxic activity, we investigated the genotoxic potential of TiO_2_ and TiO_2_/DHQ and the antigenotoxic effect of TiO_2_/DHQ. The results presented in Figure 4A,B, assessed by the comet assay, indicate the genotoxic effect of TiO_2_ and TiO_2_/DHQ in MRC-5 cells. The pristine TiO_2_ caused significant DNA damage compared to the control in the investigated concentration range (1–20 mg/mL).

Since TiO_2_/DHQ does not display genotoxicity, we investigated its antigenotoxic potential against DNA damage induced by oxidative stress. Figure 4C shows the antigenotoxic effect of TiO_2_/DHQ studied in MRC-5 cells treated with H_2_O_2_. The pretreatment of cells with TiO_2_/DHQ for 24 h resulted in a significant reduction in H_2_O_2_-induced DNA damage compared to the extent of DNA damage observed in cells exposed only to H_2_O_2_. TiO_2_/DHQ displays an antigenotoxic effect over the entire investigated concentration range (1–20 mg/L), as opposed to the ICT complex between TiO_2_ and caffeic acid, showing antigenotoxic properties at a lower concentration (0.5–1.6 mg/mL) [39]. This discrepancy is a consequence of either the different morphologies of TiO_2_ samples or, more likely, the pronounced antioxidant properties of DHQ compared to caffeic acid. The change from genotoxic to antigenotoxic behavior in TiO_2_ upon the formation of the ICT complex with DHQ suggests that DHQ retains its antioxidant properties despite the consumption of two out of its five hydroxyl groups in the condensation reaction with TiO_2_ surface hydroxyl groups.

### 2.4. Preliminary Antimicrobial Tests

**Figure 5:**
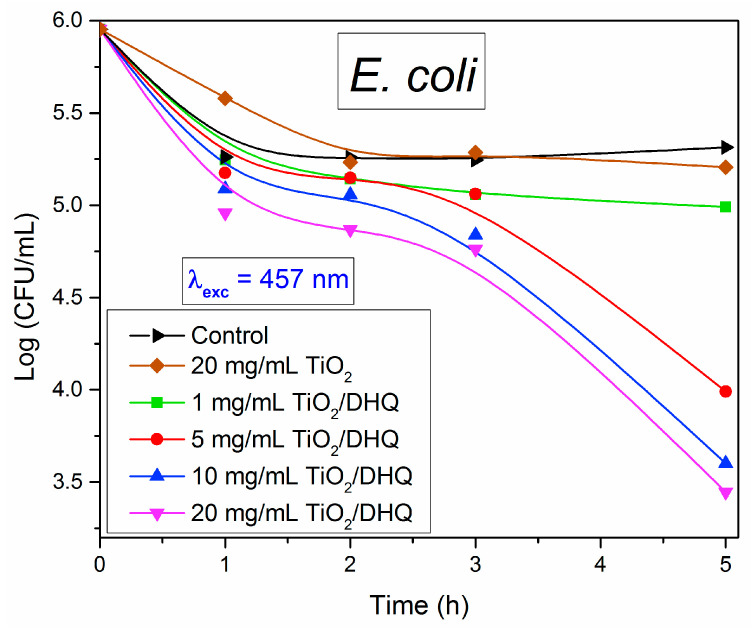
The time-dependent antimicrobial ability of pristine TiO_2_ (20 mg/mL) and TiO_2_/DHQ (1–20 mg/mL) against *E. coli* under visible-light excitation (457 nm); control: experiments without photocatalyst.

Figure 5 shows the time- and concentration-dependent antimicrobial activity of TiO_2_/DHQ against the Gram-negative bacteria *E. coli*, including data for pristine TiO_2_ (20 mg/mL) and the control (the absence of a photocatalyst). Results for two additional controls, the antibacterial activity of TiO_2_ and TiO_2_/DHQ in the dark, are presented in the Supporting Information (Table S1). Despite limited experimental data, we can discern the following conclusions. First, pristine TiO_2_ does not display antimicrobial activity against *E. coli* upon Vis light excitation; its time–kill curve practically overlaps with the control. Of course, neither of the photocatalysts (TiO_2_ and TiO_2_/DHQ) display a biocidal effect in the dark. Second, for low concentrations (1 mg/mL), TiO_2_/DHQ does not show any antibacterial properties. Third, at higher concentrations (5, 10, and 20 mg/mL), TiO_2_/DHQ demonstrates concentration-dependent antimicrobial activity. For example, after five hours of illumination, the number of surviving bacteria was reduced by two and three orders of magnitude when the TiO_2_/DHQ concentration was 5 and 20 mg/mL, respectively. Fourth, unfortunately, the kinetic curves of antimicrobial activity are incomplete since the homemade light source, designed to cover the desired spectral range, has an overheating issue, preventing continuous operation for extended periods. However, based on their trends, complete bacterial destruction can be expected under prolonged irradiation.

## 3. Discussions

### 3.1. Basic Properties of ICT Complex Between TiO_2_ and DHQ

**Scheme 1:**
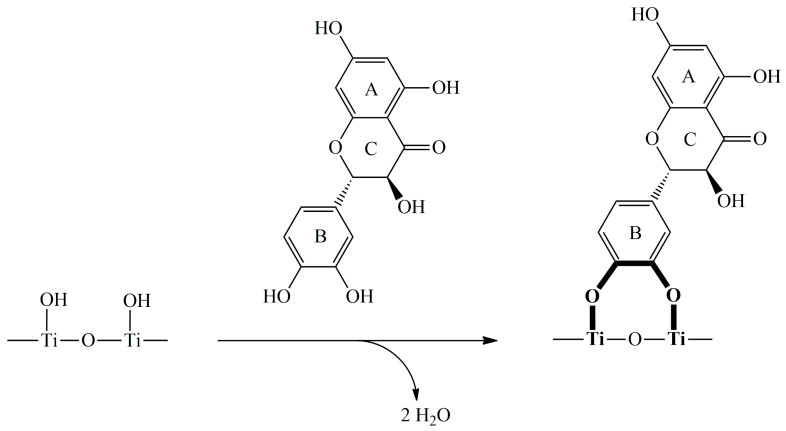
Schematic presentation of the formation mechanism of the TiO_2_-based ICT complex with DHQ.

The coordination of catechol to the TiO_2_ surface is almost intuitive since the formation of the ICT complex occurs through the condensation reaction between hydroxyl groups from the inorganic and organic components of the ICT complex. The remaining question after the infrared spectroscopy analysis of the TiO_2_–catechol complex and its components—does catechol coordinate to the TiO_2_ surface by bridging or chelating coordination—was solved by applying Job’s method of continuous variation [10], and the formation of bridging coordination was, in addition, supported by density functional calculations (DFT) [18]. Of course, due to the presence of multiple hydroxyl groups in DHQ, it is mandatory to check how DHQ coordinates to the TiO_2_ surface; i.e., is it over neighboring hydroxyl groups at positions 3′ and 4′ (ring B), with hydroxyl groups at positions 5 and 7 (ring A) and 3 (ring C) remaining free? The infrared analysis of free and bound DHQ to TiO_2_, supported by a DFT calculation using the [Ti_18_O_31_(OH)_8_]/DHQ cluster as a model system, undoubtedly proved the above-mentioned coordination of DHQ to the TiO_2_ surface [29]. For clarity, the formation mechanism of TiO_2_/DHQ, based on the condensation reaction, and the surface structure of the ICT complex are presented in Scheme 1.

The way in which DHQ binds to the TiO_2_ surface significantly affects the bioactivity of the ICT complex since hydroxyl groups on the phenolic ring display antioxidant activity. The presence of multiple hydroxyl groups increases the ability of the phenolic compound to donate hydrogen atoms to reactive free radicals, stabilizing them. In addition, the aromatic ring of phenolic compounds increases their antioxidant activity. So, on the one hand, although two hydroxyl groups at positions 3′ and 4′ (ring B) are consumed in the formation of the Ti–O–C linkage between TiO_2_ and DHQ, the remaining three free hydroxyl groups can significantly reduce the toxicity of TiO_2_. On the other hand, although the TiO_2_-based ICT complex with DHQ should have increased photoinduced antimicrobial performance due to the visible-light response, the ability of free hydroxyl groups to scavenge ROS can somewhat diminish its antimicrobial ability.

### 3.2. Cytotoxicity

The functionalization of TiO_2_ with DHQ increased the cytotoxic potential of cervical cancer cells, consistent with previous findings of DHQ cytotoxicity in HeLa cells [40]. It should be mentioned that the inhibitory effect on cell viability was more pronounced in HeLa cancer cells than in normal MRC-5 cells, which is most likely a consequence of different DHQ effects in cancer versus normal cells. In a recent study, Mohammed et al. [41] indicated that DHQ and its metabolites have better inhibitory activity on the colon HCT-116 cancer cell line than on HEK-293 normal cells in the tested concentration range. Further, a study by Chahardoli et al. [42] showed that biosynthesized TiO_2_ nanoparticles using quercetin had dual effects in normal and cancer cells, with IC50 values below 100 and 50 μg/mL for human breast cancer cells of the MCF-7 cell line and melanoma cancer cells of the A375 cell line, respectively. However, biosynthesized TiO_2_ nanoparticles did not significantly affect normal skin fibroblast cells at up to 50 μg/mL.

In conclusion, the TiO_2_-based ICT complex with DHQ has increased antioxidative potential compared to pristine TiO_2_ in both types of cells, and this observation is in accordance with related studies. Previous results indicate that DHQ inhibits intracellular ROS generation in human cells in a dose-dependent manner, showing the pronounced inhibition of ROS generation with an increase in DHQ concentration from 10 to 100 µg/mL [43]. Also, DHQ displays a reduction in the activity of antioxidant enzymes (superoxide dismutase (SOD), catalase (CAT), and glutathione peroxidase (GPX)) in pretreated cells, along with decreased intracellular ROS production [44]. Li et al. [45] observed that DHQ can efficiently scavenge ^•^OH, DPPH^•^, and ABTS^•+^ (hydroxyl, 2,2-diphenyl-1- picrylhydrazyl, and 2,2′-azino-bis (3-ethylbenzothiazoline-6-sulfonic acid) diammonium salt radicals, respectively) and protect bone marrow-derived mesenchymal stem cells from ^•^OH-induced cytotoxicity. Also, DHQ prevented oxidative damage, such as ROS generation, glutathione depletion, and single-strand break formation, in human primary dermal fibroblasts (NHDFs) and epidermal keratinocytes (NHEKs) [46]. In addition, in a previous study, we showed that free hydroxyl groups at positions 3, 5, and 7 of the DHQ moiety in TiO_2_/DHQ enable a reduction in the ABTS^•+^ radical cation, while exposure to UV light further enhances this reduction. In the presence of TiO_2_, photogenerated electrons reduce ABTS^•+^, whereas in the presence of TiO_2_/DHQ, both photogenerated electrons and hydroxyl groups contribute to the decrease in ABTS^•+^, enabling the ICT complex to exhibit both photocatalytic and radical-scavenging properties. Also, TiO_2_ did not affect the DPPH radical concentration, while TiO_2_/DHQ induced a 35% decrease in the relative DPPH concentration due to the radical-scavenging potential of the hydroxyl groups on the DHQ moiety attached to the TiO_2_ surface [29].

Based on the results of this study and the literature data, the TiO_2_-based ICT complex with DHQ has increased cytotoxicity and a pronounced antioxidant effect compared with pristine TiO_2_ due to DHQ’s antioxidant properties. This conclusion agrees with a report showing that titanium dioxide nanotubes loaded with quercetin are more effective anticancer and ROS-reducing agents than titanium dioxide nanotubes or quercetin themselves [47].

### 3.3. Genotoxicity and Antigenotoxicity

As already shown, TiO_2_ nanoparticles can cause adverse reactions, leading to cell damage, inflammation, and immune responses [48]. The comet assay, used in this study, is commonly used in the genotoxicity testing of DNA damage and repair in various experimental models [49]. Several research groups have already indicated the potential risks of genotoxicity associated with exposure to TiO_2_ nanoparticles, showing that they can cause DNA and chromosome damage and gene mutations either in in vitro or in vivo experiments [50,51,52]. The form and degree of damage highly depend on the physical and chemical properties of TiO_2_, which govern its reactivity and bioavailability [48]. Its genotoxic effects do not only depend on the morphology of TiO_2_ particulates (size and shape) but also on the test subjects (cells/animals) and the type, concentration, and duration of exposure [48,50,51,52]. We showed that pristine TiO_2_ can cause significant DNA damage in MRC-5 cells. Although three mechanisms are recognized for the genotoxic effects of TiO_2_ particulates, ranging from nano- to micro-sizes, the available data suggest that oxidative stress, e.g., nanoparticle-induced ROS production, is a signal of other physiological effects, including cytotoxicity and genotoxicity [50,53].

However, the TiO_2_-based ICT complex with DHQ did not induce DNA damage in MRC-5 cells under the same experimental conditions (Figure 4B). These results are consistent with the literature data obtained for ICT complexes between nanometer-sized TiO_2_ particles with ascorbic acid [31] and caffeic acid [39], showing no DNA damage in leukocytes from whole blood cells (0.4–8.0 mg/mL). Also, the ICT complex between TiO_2_ powder (Degussa P25), the same one used in this study, and caffeic acid did not induce DNA damage when orally administered to mice (2000 mg/kg) [33].

The change from genotoxic to antigenotoxic behavior in TiO_2_ upon the formation of the ICT complex with DHQ suggests that DHQ retains its antioxidant properties despite the consumption of two out of its five hydroxyl groups in the condensation reaction with TiO_2_ surface hydroxyl groups.

### 3.4. Preliminary Antimicrobial Tests

Particulate matter’s growing applications in various fields have raised concerns about its toxicity, and therefore, the use of functionalized TiO_2_ particles with DHQ is advantageous from an ecological perspective. An additional advantage of TiO_2_/DHQ is its enhanced harvesting ability for solar radiation. While many studies have demonstrated the antimicrobial activity of TiO_2_ under UV light [2,3,4,5,7,8], even though ICT complexes are eco-friendly and Vis light sources can be used instead of harsh UV light sources, the literature on the biocidal action of ICT complexes under Vis light is almost non-existent [30]. So, in this study, we performed a preliminary antimicrobial test of the TiO_2_-based ICT complex with DHQ under Vis light excitation (457 nm is the maximum emission wavelength of the light source; see Figure 1) against *E. coli* to estimate if this system is worth further investigation in this direction.

The presented results are a step forward in the design of a non-toxic antimicrobial agent. Owing to free hydroxyl groups, the coordination of DHQ to the TiO_2_ surface confers antioxidant behavior to the entire ICT architecture, alongside enhanced optical properties. However, since free hydroxyl groups diminish the antimicrobial potency of the ICT complex, further research in this area should focus on increasing the efficiency of antimicrobial activity while keeping the ICT complex non-toxic.

## 4. Material and Methods

### 4.1. Synthesis and Optical Characterization of ICT Complex Between TiO_2_ and DHQ

Dihydroquarcetin (DHQ; commercial product of SIBPRIBOR OOO, Irkutsk, Russia, 97% purity) was obtained thanks to Professor Lada Živković (Faculty of Pharmacy, University of Belgrade). TiO_2_ (Aeroxide^®^ P25) was purchased from ACRŌS ORGANICS (Geel, Belgium). All other used chemicals were high-grade (Sigma-Aldrich, St. Louis, MO, USA). Milli-Q deionized water with a resistivity of 18.2 MΩ cm^−1^ was used as a solvent.

The TiO_2_-based ICT complex with DHQ was prepared following the procedure described in our recent publication [29]. Briefly, 0.5 g of TiO_2_ powder was combined with 317 mg of DHQ dissolved in 250 mL of deionized water. The dispersion was vigorously stirred for 3 h at 40 °C and left without stirring at room temperature for another 48 h. The powder was almost immediately colored, indicating the formation of the ICT complex. Then, the solid was separated by centrifugation, thoroughly washed five times with 20 mL of water, and dried in the oven at 40 °C.

Reflection spectroscopy measurements of pristine TiO_2_ and TiO_2_/DHQ were carried out using a Shimadzu UV−Visible UV-2600 spectrophotometer (Kyoto, Japan) equipped with an integrated sphere (ISR-2600 Plus, Kyoto, Japan). The content of DHQ in the TiO_2_/DHQ sample was determined by thermogravimetric analysis (TGA) using a Setaram Setsys Evolution-1750 instrument, Austin, TX, USA. The measurements were performed from room temperature to 700 °C at a heating rate of 10 °C/min in a dynamic air atmosphere with a flow rate of 20 cm^3^/min. Detailed information concerning composition, based on thermogravimetric analysis, surface structure, i.e., the coordination of DHQ to the surface of Ti atoms, based on infrared measurements, and the energy alignment of TiO_2_/DHQ, based on DFT calculations, can be found in reference [29].

### 4.2. Biological Effects of ICT Complex Between TiO_2_ and DHQ

#### 4.2.1. Treatment Preparation

TiO_2_ and TiO_2_/DHQ stock solutions (40 mg/mL) were prepared by suspending powders in a complete RPMI medium (RPMI 1640 medium (Biowest, Nuaillé, France), 10% fetal calf serum (FCS, Gibco, Waltham, MA, USA), and 1% antibiotic–antimycotic solution (Capricorn Scientific GmbH, Ebsdorfergrund, Germany)). The suspensions were left for 24 h in a humified incubator with 5% CO_2_ at 37 °C to allow for the release of any material attached to the TiO_2_ surface. After 24 h, the supernatant was separated from the solid by centrifugation at 3000× *g* for 10 min. Then, the supernatant was diluted with fresh RPMI medium in serial dilutions to reach final concentrations (1, 2, 5, 10, and 20 mg/mL) to be used in further experiments.

#### 4.2.2. Cell Culture

Human fetal lung fibroblasts (MRC-5) and human cervical carcinoma cells (HeLa) were propagated in 25 cm^2^ tissue culture flasks in a humidified incubator with 5% CO_2_ at 37 °C. MRC-5 and HeLa cells were grown in a complete medium. After reaching 70% confluence, the cells were trypsinized (0.25% trypsin–EDTA solution, Institute for Virology, Vaccines, and Sera “Torlak”, Belgrade, Serbia), seeded in 96-well plates (1.5 × 10^4^ cells/well), and left to attach to wells for 24 h at 37 °C (5% CO_2_)

#### 4.2.3. Cytotoxicity Evaluation

The MRC-5 and HeLa cells were treated with TiO_2_ and TiO_2_/DHQ in the concentration range described in Section 4.2.1. (total volume of 100 µL per well). Following incubation with the treatments or medium alone (control) at 37 °C for 24 h, we performed an MTT assay. MTT reagent at a working concentration of 0.5 mg/mL (thiazolyl blue tetrazolium bromide, Sigma Aldrich, St. Louis, MO, USA) was added (10 µL per well), and the cells were left for 2 h in the dark at 37 °C for the reaction to occur. Then, purple formazan crystals were dissolved with sodium dodecyl sulfate (10% SDS in 0.01 M HCl, Sigma Aldrich, St. Louis, MO, USA), and the absorbance was measured at 570 nm on a microplate reader (BioTek ELx800, Winooski, VT, USA) after the complete solubilization of the crystals. Three independent experiments in triplicate were performed (*n* = 9).

#### 4.2.4. H2DCFDA Assay (2′, 7′-Dichlorofluorescin Diacetate)

To evaluate ROS production in MRC-5 and HeLa cells, the medium in the cell cultures, prepared according to the procedure outlined in Section 4.2.2., was exchanged with 100 μL/well treatments with various concentrations of TiO_2_ and TiO_2_/DHQ prepared as described in Section 4.2.1. After 24 h, treatments were removed, and cells were rinsed with PBS. Further, we followed the manufacturer’s instructions for the DCFDA assay. So, 5 μM of the cell-permeable oxidation-sensitive probe, H2DCFDA (Merck Millipore, Burlington, MA, USA, 2′,7′-Dichlorofluorescin Diacetate-CAS 4091-99-0-Calbiochem), using PBS as the diluent, was added to the cells and left for 45 min in the dark. Next, the cells were washed with PBS and exposed to PBS alone (control) or 200 μM of H_2_O_2_ (positive control). After 1 h of incubation and the conversion of non-fluorescent H2DCFDA to the highly fluorescent 2’,7’-dichlorofluorescein (DCF), the intracellular ROS generation level in cells was determined by measuring the fluorescence at 535 nm upon excitation at 485 nm using a fluorescent plate reader (Wallac 1420 multilabel counter Victor 3V, Perkin Elmer, Shelton, CT, USA). Data were expressed as relative fluorescence intensity. Three independent experiments in triplicate were performed (*n* = 9).

### 4.3. Comet Assay

Before processing for the alkaline comet assay, MRC-5 cells were incubated for 24 h with TiO_2_ or TiO_2_/DHQ at a range of concentrations in a complete RPMI medium in 96-well plates for genotoxicity testing. For the antigenotoxic effect, after rinsing with PBS, 50 µM of H_2_O_2_ was added to a serum-free medium to induce DNA damage in the 24 h-incubated cells with TiO_2_/DHQ. After the incubation period, the treatments were rinsed with PBS. Then, the cells were trypsinized with a 0.25% trypsin–EDTA solution and centrifuged at 300× *g* for 5 min. The cell pellets were resuspended in a complete medium to achieve a density of approximately 1 × 10^4^ cells.

The comet assay was performed according to Tice et al. [54]. Cells were suspended in 0.5% low-melting-point agarose (Sigma Aldrich, St. Louis, MO, USA) and pipetted onto slides pre-coated with 1% normal-melting agarose (Sigma Aldrich, St. Louis, MO, USA). The slides were covered with coverslips and stored at 4 °C for 5 min to solidify the agarose. After removing the coverslips, the slides were immersed in a pre-cooled lysis solution (2.5 M NaCl, 100 mM EDTA, 10 mM Tris, 1% Triton X-100, and 10% dimethyl sulfoxide; pH 10, adjusted by NaOH) and stored overnight at 4 °C.

The next day, the slides were placed in electrophoresis buffer in a horizontal tank, and electrophoresis was conducted at 4 °C for 30 min at a constant voltage (0.8 V/cm). After electrophoresis, the slides were neutralized and stained with ethidium bromide (20 µg/mL). The comets were examined under an Olympus BX 50 microscope (Olympus Optical Co., GmbH, Hamburg, Germany), equipped with a mercury lamp HBO (50 W, 516–560 nm, Zeiss, Oberkochen, Germany). Comets were visually scored and classified into five categories (A, B, C, D, and E) based on the extent of DNA damage, as described by Anderson et al. [55]. The degree of DNA damage was expressed as the mean number of cells in comet classes B + C + D + E. Two replicate slides were prepared for each treatment, and 100 randomly selected cells per slide were analyzed. The entire experiment was repeated three times.

### 4.4. Antimicrobial Activity

The photoinduced bactericidal activity of TiO_2_/DHQ was evaluated against the Gram-negative bacteria *E. coli* (ATCC 25922) under nearly monochromatic Vis light illumination (457 nm, see Figure 1). The microbial inoculum was prepared by transferring the microorganisms into 3 mL of tryptone soy broth (TSB) supplemented with 0.6% yeast extract (TSB1Y) and incubating them overnight at 37 °C (~18 h). The incubated test cultures were diluted to a working concentration of approximately 10^5^ CFU/mL.

Various amounts of the TiO_2_/DHQ samples (25, 50, and 100 mg) were placed in glass Petri dishes containing 5 mL of the microbial suspension in saline solution (8.5% NaCl). The Petri dishes were incubated under the light for 5 h. After a series of dilutions, a 0.1 mL aliquot of the solution was transferred into a sterile Petri dish and overlaid with tryptone soy agar (1.5% agar in TSB1Y). The inoculated plates were incubated at 37 °C for 24 h, and the surviving cells were counted (CFU/mL; CFUs—colony-forming units).

To obtain unambiguous information about the photocatalytic antibacterial activity of TiO_2_/DHQ under exclusive Vis light excitations, we performed the following control experiments: the time-dependent reduction of *E*. coli under Vis light illumination in the absence of any photocatalyst, the time-dependent reduction of *E*. coli in the presence of TiO_2_/DHQ in the dark, and the time-dependent reduction of *E*. coli in the presence of pristine TiO_2_ in the dark, as well as under Vis light illumination.

## 5. Conclusions

In conclusion, we prepared surface-modified TiO_2_ with a potent plant-derived antioxidant dihydroquercetin (DHQ) and assessed its bioactivity. Besides inducing red-shifted absorption due to ICT complex formation, the coordination of DHQ to the surface of Ti atoms reduced the genotoxicity of TiO_2_ generated by reactive oxygen species (ROS) formed upon the excitation of the hybrid architecture. We demonstrated that the TiO_2_-based ICT complex with DHQ is non-toxic to MRC-5 cells up to a concentration of 10 mg/mL and exhibits significant antigenotoxic properties compared with pristine TiO_2_ nanopowder. Furthermore, preliminary antibacterial tests indicated that the TiO_2_-based ICT complex with DHQ efficiently inactivates the Gram-negative bacteria *E. coli* when exposed solely to Vis light. We are confident that further research in this direction is worthwhile, considering that, on the one hand, expensive and harmful UV light sources are replaceable by cost-effective, harmless Vis light sources, while, on the other hand, the release of toxic materials, for example, silver, into the environment can be avoided.

## Data Availability

The data presented in this study are available on request from the corresponding author.

## Supplementary Materials

The following supporting information can be downloaded at https://www.mdpi.com/article/10.3390/ijms26041475/s1.
