# Supplementary material for: Bioactivity Assessment of Functionalized TiO2 Powder with Dihydroquercetin
Source: Int J Mol Sci. 2025 Feb 10;26(4):1475. doi: 10.3390/ijms26041475 (PMC11855565; doi:10.3390/ijms26041475)

Supporting Information

Bioactivity Assessment of Functionalized TiO_2 Powder with Dihydroquercetin

Valentina Nikšić [1], Andrea Pirković [2], Biljana Spremo-Potparević [3], Lada Živković [3], Dijana Topalović [3], Jovan M. Nedeljković [1] and Vesna Lazić [1,*]

1 Vinča Institute of Nuclear Sciences—National Institute of the Republic of Serbia, Centre of Excellence for Photoconversion, University of Belgrade, 11351 Belgrade, Serbia; valentina.niksic@vin.bg.ac.rs (V.N.); jovned@vin.bg.ac.rs (J.M.N.)

2 Department for Biology of Reproduction, INEP Institute for Application of Nuclear Energy, University of Belgrade, 11080 Belgrade, Serbia; andrea.pirkovic@inep.co.rs

3 Department of Pathobiology, Faculty of Pharmacy, University of Belgrade, 11221 Belgrade, Serbia; bilja22@pharmacy.bg.ac.rs (B.S.-P.); lada@pharmacy.bg.ac.rs (L.Ž.); dijana@pharmacy.bg.ac.rs (D.T.)

* Correspondence: vesna.lazic@vin.bg.ac.rs

Table S1. Antimicrobial efficiencies of pristine TiO_2 and TiO_2/DHQ against *E. coli* as a function of time in the dark.

		**Time (h)**				
		0	1	2	3	5
**Log (CFU/mL)**	**pristine TiO_2 (20 mg)**	5.079	5.000	5.079	4.531	4.748
	**TiO_2/DHQ (20 mg)**	5.079	4.914	4.833	5.041	5.079

Figure S1. The TG curve of the TiO_2/DHQ sample in air.

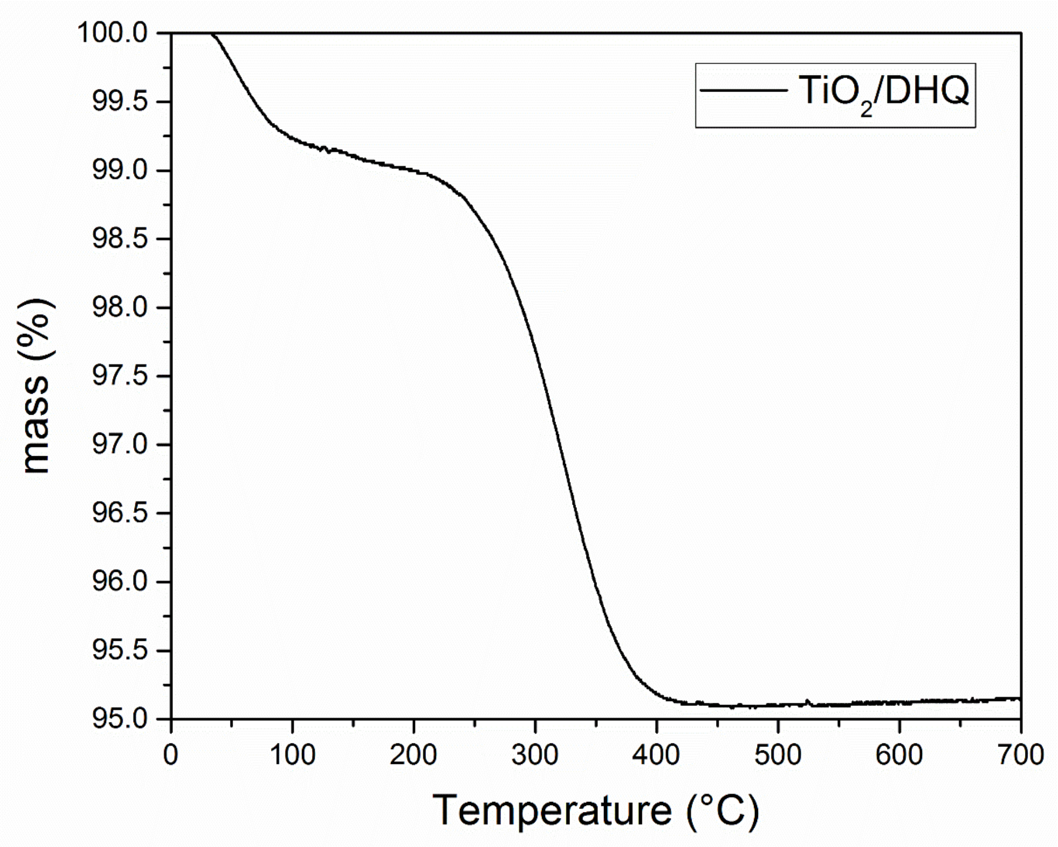

Supplement: Supplementary file 1 [file ijms-26-01475-s001.zip › ijms-3458068-supplementary.pdf]
